# France’s State of the Art Distributed Optical Fibre Sensors Qualified for the Monitoring of the French Underground Repository for High Level and Intermediate Level Long Lived Radioactive Wastes

**DOI:** 10.3390/s17061377

**Published:** 2017-06-13

**Authors:** Sylvie Delepine-Lesoille, Sylvain Girard, Marcel Landolt, Johan Bertrand, Isabelle Planes, Aziz Boukenter, Emmanuel Marin, Georges Humbert, Stéphanie Leparmentier, Jean-Louis Auguste, Youcef Ouerdane

**Affiliations:** 1 National Radioactive Waste Management Agency (Andra), F-92298 Chatenay-Malabry, France; marcel.landolt@andra.fr (M.L.); johan.bertrand@andra.fr (J.B.); isabelle.planes@univ-st-etienne.fr (I.P.); stephanie.leparmentier@xlim.fr (S.L.); 2 Laboratoire Hubert Curien CNRS UMR 5516, University of Lyon, F-42000 Saint-Etienne, France; sylvain.girard@univ-st-etienne.fr (S.G.); aziz.boukenter@univ-st-etienne.fr (A.B.); emmanuel.marin@univ-st-etienne.fr (E.M.); ouerdane@univ-st-etienne.fr (Y.O.); 3 XLIM Research Institute, UMR 7252 CNRS/University of Limoges, 123 Avenue Albert Thomas, F-87060 Limoges, France; georges.humbert@xlim.fr (G.H.); auguste@xlim.fr (J.-L.A.)

**Keywords:** optical fibres, optical fibre sensors, Raman, Brillouin, Rayleigh scatterings, temperature, strain, radiation effects, hydrogen

## Abstract

This paper presents the state of the art distributed sensing systems, based on optical fibres, developed and qualified for the French Cigéo project, the underground repository for high level and intermediate level long-lived radioactive wastes. Four main parameters, namely strain, temperature, radiation and hydrogen concentration are currently investigated by optical fibre sensors, as well as the tolerances of selected technologies to the unique constraints of the Cigéo’s severe environment. Using fluorine-doped silica optical fibre surrounded by a carbon layer and polyimide coating, it is possible to exploit its Raman, Brillouin and Rayleigh scattering signatures to achieve the distributed sensing of the temperature and the strain inside the repository cells of radioactive wastes. Regarding the dose measurement, promising solutions are proposed based on Radiation Induced Attenuation (RIA) responses of sensitive fibres such as the P-doped ones. While for hydrogen measurements, the potential of specialty optical fibres with Pd particles embedded in their silica matrix is currently studied for this gas monitoring through its impact on the fibre Brillouin signature evolution.

## 1. Introduction

In the Cigéo project, the French underground repository for high-level and intermediate level long-lived radioactive wastes program, monitoring in the civil engineering structures used and host rock is planned in order to confirm the long-term safety evaluation and recoverability of wastes during the exploitation period. Work is in progress in order to select the exact parameters to monitor. Andra, the French national agency for radioactive waste management, supports sensor developments and qualification tests for the most relevant processes that will be developed during Cigéo implementation, namely thermal, hydraulic, mechanical, chemical and radioactive (T-H-M-C-R).

To ensure long-term monitoring despite harsh conditions, several and diverse monitoring technologies are considered and would be combined inside the repository cells: both non-destructive evaluation and embedded sensors, as well as electronic and optical sensors, including direct and indirect measurements of parameters of interest, mixing traditional and innovative sensing technologies.

Optical fibre sensors (OFS) are found to be exceptional tools, as they enable distributed measurements, thus providing data over the entire structure. Monitoring with a single fibre can provide information all along the structure behavior, and thus overcome limitations of traditional sensors, whose information is restricted to local effects. Moreover, optical fibre’s small size enables one to reduce invasiveness. Remote sensing would enable the maintenance of the optoelectronic devices during the facility lifetime; only the optical fibre, that is known to be more resistant than electronics, can be exposed to the harsh conditions during the operating period. This explains why, since 2006, Andra has deployed a large qualification process on distributed optical fibre sensing systems in collaboration with several companies and research laboratories.

Optical fibres are known and widely spread in industry to provide strain and temperature measurements. Yet they must be designed to resist to the specific environmental conditions of Cigéo: presence of hydrogen, relatively high temperature (100 °C) and gamma rays. This article presents the target application in a first part (Section 2), and the qualification methodology in a second part (Section 3). State of the art temperature sensing is presented in Section 4, while strain sensing is detailed in Section 5, hydrogen and radiation sensing are reported in Section 6 and Section 7, respectively. The updated knowledge is quoted in Technology Readiness levels (TRL, [1]). This overview includes developments supported by Andra in France or reported in literature by other French labs.

## 2. The Target Application: Monitoring the Underground Repository of High Level and Intermediate Level Long-Lived Radioactive Wastes

### 2.1. Repository Cells

Cigéo is designed to isolate the radioactive waste from humans and the biosphere and to confine it within a deep geological formation to prevent dissemination of the radionuclides contained in this waste. In the French design, the disposal takes placed in a clay formation, an argillaceous rock (clay rock) known as the Callovo-Oxfordian formation, which is approximately 155 million years old and lies at a depth of 400 to 600 m. 

**Figure 1:**
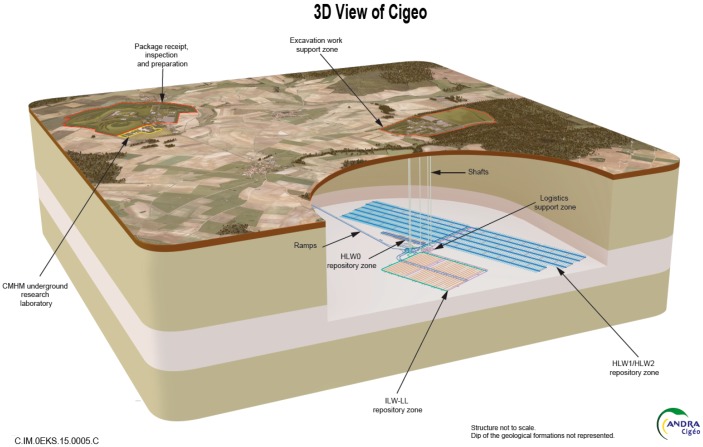
Preliminary design of Cigéo, the underground repository for high level and intermediate level long-lived radioactive wastes.

As sketched in Figure 1, the underground facility has separate areas designed for high-level waste (HLW) and intermediate-level long-lived waste (ILW-LL) in order to limit phenomenological interactions between these different waste categories. The cells are spherical or ovoid underground excavations on a horizontal axis or slight slope dug out of the Callovo-Oxfordian formation. There are two categories of cells. HLW micro-tunnel cells would contain only one disposal package per cell section; with main dimensions of 0.7 m diameter, 100 m long, and a metallic liner. ILW-LL tunnel cells would contain several disposal packages per cell section (in the order of 9 m size diameter). Their mechanical stability during operation is guaranteed by a concrete liner, left in place at closure. Length would be on the order of 600 m.

### 2.2. Monitoring Objectives

An appropriate level of monitoring and control will be applied to Cigéo during its construction and its operation, to ensure the protection and preservation of the passive post-closure safety features. Observation and surveillance parameters are not fully stated yet. A global analysis based on safety analysis, technology readiness level [1] of monitoring technologies and risks is currently performed within Andra, and also at the European level within the MoDeRn and MoDeRn2020 European projects [2,3].

OFS are mainly envisioned for temperature and mechanical evaluations. Temperature monitoring contributes to the assessment confirmation basis for long term safety, by quantifying margins of prior predictions for the duration of the thermal period and the expected thermal peaks. Mechanical parameters are also required, to confirm that the waste recoverability is possible, if decided; indeed repository reversibility is stated by the French Planning Act No. 2006-739 dated 28 June 2006 on the sustainable management of radioactive waste.

To provide accurate and verified measurements, it is also important to characterize all parameters influencing the sensing chain performances. This is why hydrogen and radiation also appear in the target monitoring parameters of Table 1. Gamma radiation dose rate ranges from 1 to 10 Gy(SiO_2_)/h depending on the waste category. Over the target lifetime of 100 years, the sensors could experience ionizing doses up to 10 MGy(SiO_2_). The atmosphere will become hydrogen-rich because H_2_ emissions originating from anoxic corrosion of metallic materials or some specific radioactive IL waste release hydrogen (cf. [2]). Measuring either hydrogen or oxygen (related to corrosion speed) or both would assess chemical evolutions.

Possible target monitoring parameters, expected sensing range and uncertainties are detailed in Table 1. As most phenomena kinetics are slow, their corresponding measurement frequencies can be selected on a daily basis.

As the parameters are not homogeneously distributed around the repository cells, it is important to develop sensors able to perform truly distributed sensing, to characterize a spatial mapping distribution.

### 2.3. Envisionned Implementation of OFS in Repository Cells

**Figure 2:**
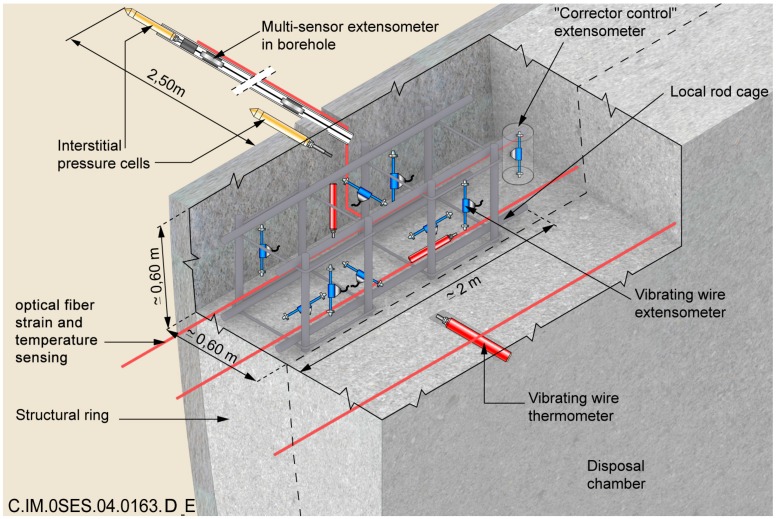
Envisioned monitoring system for ILW-LL repository cells.

In the reference monitoring system design, sensors are embedded in concrete liners of Intermediate—level (IL) repository cells, as sketched in Figure 2, or on the external surface of the metallic liner of High-Level (HL) repository cells. Regarding the application, more details can be found in [2].

### 2.4. Environmental Conditions in the Repository Cells

Improving and hardening the sensor radiation tolerance is a major issue for the design of reliable, long-term equipment. Worst case will be considered for the qualification process performed by Andra, namely the vicinity of HLW repository cell. At the extreme conditions, humidity will reach 100%, hydraulic pressure 6 MPa, lithostatic pressure 12 MPa, dose rate 1 Gy/h and temperature 90 °C.

## 3. Materials and Methods

### 3.1. Qualification Method

**Figure 3:**
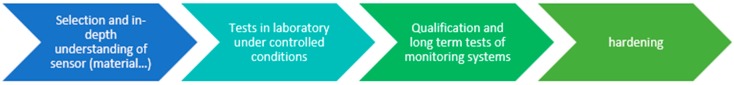
Sensing chain qualification methodology.

To fulfill these challenging application requirements, several monitoring technologies are considered and would be used in combination inside the repository cells. For each sensing chain, a qualification method in several steps is deployed. First we select the most promising technologies which are tested in controlled situations. If these tests are successful, the sensor can be implemented into real structures, to quantify its impact, integration and response in representative environments. A last step is devoted to the harsh environments vulnerability study: accelerated aging tests, for instance radiation exposure, are organized. This methodology is summarized in Figure 3.

In the present review, we will summarize and point out developments realized in France by Andra and its research partners to qualify a sensing chain and provide truly distributed measurements of temperature, strain, hydrogen and radiation dose levels.

### 3.2. Choice of Sensing Principle—Scatterings in Optical Fibres

Distributed sensing provides a versatile and powerful monitoring tool. The term distributed optical fibre sensor designates the case in which the silica-based material becomes a sensor. It is thus no longer necessary to implement anticipated sensor positions since measurements are being performed all along the optical fibre connected to the probe/reading device (as well as within the extension cables). Truly distributed measuring systems for temperature and strain sensing were transposed from labs to industrial applications in 2000 s. Instruments combine (i) a sensitive phenomenon based on Brillouin, Rayleigh or Raman scatterings with (ii) a localization process, usually Optical Time Domain Reflectometry (OTDR), Optical Frequency Domain Reflectometry (OFDR, as in Luna-OBR device mentioned later) or coherent probe-pump techniques (as in the Neubrescope device mentioned later).

Rayleigh and Brillouin scatterings are sensitive to both strain and temperature whereas Raman scattering depends only on temperature. More precisely, the Raman Anti-Stokes intensity is significantly more sensitive to temperature than the Stokes component. Most commercial devices determine the optical fibre temperature from the spectral analysis of the scattered light, by computing the ratio between the Anti-Stokes and Stokes intensities from the Equation (1) [4]:

(1) $$\frac{I_{\mathrm{AS}}}{I_{S}}=\;\frac{{\left(\upsilon_{0}+{\Delta \upsilon }_{\mathrm{Raman}}\right)}^{4}}{{\left(\upsilon_{0}-{\Delta \upsilon }_{\mathrm{Raman}}\right)}^{4}}\;.{\;e}^{-\frac{h\;.{\Delta \upsilon }_{\mathrm{Raman}}}{K_{B}.T}}$$

where I_AS_ and I_S_ are the Anti-Stokes and Stokes intensities respectively, h is Planck’s constant (given as 6.62607004 × 10^−34^·m^2^·kg·s^−1^), Δυ_Raman_ is the Raman frequency shift (13.2 THz for silica) and υ_0_ is the probe laser pulse frequency (in Hz), K_B_ is the Boltzmann constant (given as 1.38064852 × 10^−23^ m^2^·kg·s^−2^·K^−1^) and T the absolute temperature (in Kelvin, K).

The Brillouin frequency shift Δυ_B_ is known to be proportional to temperature and strain (ɛ) variations [5], so it can be defined as:

Δυ_B_ = C_ɛ_^B^ε + C_T_^B^ ΔT (2)

where C_T_^B^ and C_ɛ_^B^ are the sensitivity coefficients for both temperature and strain, respectively.

If measured at a single wavelength, Rayleigh scattering provides only a detection of events along an optical fibre. To supply temperature and strain measurements, commercial devices exploit the temperature and strain sensitivities of the Rayleigh backscattered intensity over a spectral window. Two measurements should be acquired and correlated, this technique being able to follow the strain or temperature changes occurring with respect to an initial condition considered as a reference. The scattered profiles from the two datasets are cross-correlated along the perturbed portion of the fiber to obtain the spectral shift in this part of the fiber caused by temperature and /or strain change. We call this change of the correlation peak position “Rayleigh spectral shift”, Δυ^R^. It is related to both strain and temperature via calibration coefficients C_T_^R^ and C^R^_ε_ [6]:

Δυ^R^ = C_ɛ_^R^ε + C_T_^R^ ΔT, (3)

Detailed description of s truly distributed sensing scheme is available in [7] and more recently in [8]. Performances are summarized in Table 2 for comparison. Values are orders of magnitude; performances may significantly change with the operation wavelength, the device supplier, the sensing optical fibre type... Raman device performances should reach 0.1 °C uncertainty over 30 km, with a length resolution of 1 m. Brillouin systems provide 1 °C or 20°·µm/m uncertainties, over 30 km, with a spatial resolution of 1m. Rayleigh scattering sensing devices exhibit higher performances than Brillouin based systems. Depending on the localization process, there is a trade-off to find between the maximal sensing range and the spatial resolution. For OFDR [9] (and tunable-OTDR, respectively [10]) the measurement distance range is 70 m (or 20 km, respectively), and spatial resolution in the order of 1 cm (resp. 20 cm). It is worth noticing that both Rayleigh and Brillouin instruments are using single-mode fibres (SMF) and operate at wavelength around 1.55 µm. For distance range smaller than 20 km, most commercially-available Raman devices use multi-mode fibres (MMF) at around 1064 nm. It implies that the three device types cannot be paired with the same optical fibre. Raman singlemode devices exist but are less efficient on short distance consistently with [11] (and more expensive).

Performances summarized in Table 2 are obtained with optical fibres in laboratories and would be significantly degraded in real application. More precisely, although Andra’s application is limited to the kilometer range, with the high excess of optical losses induced by radiations, optical budget would reach 10 dB after a propagation length of 1 km only. One should wonder whether short distance with high losses equals long distance and low-loss, on the sensing performance point of view.

Because of the complementary performances, of these various techniques for strain and temperature distributed sensing, qualification procedure has been realized on the sensors exploiting the three scattering processes.

Influences of hydrogen and radiations presence on strain and temperature distributed sensing were poorly known in 2008 when Andra started its qualification process. The first studies were devoted to the vulnerability of those technologies to radiations. It was demonstrated that the sensors based on the three scattering processes are significantly influenced by: (i) radiation; (ii) hydrogen diffusion into the optical fibre and (iii) their coupled effects. The main results of these analyses as the outcomes of the next projects devoted to the hardening of these technologies are discussed in the following sections of this review (Section 3.8 and Section 3.9). Recent works and related methodologies for radiation and hydrogen sensing are detailed in sections Section 6 and Section 7, respectively.

### 3.3. Optical Fibre Choice/Design: Dopant and Primary Coatings

Claimed performances are obtained with standard telecommunication fibres, usually single-mode G652 type for temperature sensing, G657 type for strain sensing based on Brillouin or Rayleigh scatterings, graded-index 50 µm core multi-mode fibre for temperature sensing based on Raman scatterings. 

These properties might not be preserved under harsh environmental conditions of Cigéo. Optical fibres darken under radiation through a phenomenon called Radiation-Induced Attenuation (RIA). The amplitudes and kinetics of these excess losses depend on many parameters including the fibre composition and the operation wavelength. Standard telecommunication fibres usually possess Ge-doped core and various possible cladding compositions. Both core and cladding dopants strongly impacts the fibre radiation response [12]. They are known to handle low gamma irradiation doses. For harsh environments such as Cigéo, pure silica core fibres with F dopants in the optical claddings have been developed [13]. On the opposite, P and Al dopants are used to obtain radiation sensitive fibres [14]. This is why Andra’s qualification procedure has been implemented with this variety of optical fibres, from the highly sensitive fibre to the most resistant one. More precisely, Andra’s partners, Laboratoire Hubert Curien, (LabHC—Université de Lyon, CNRS UMR 5516), selected a large variety of optical fibres. Samples whose results are presented in the article are detailed in Table 3.

Regarding primary coatings, acrylate is used for telecommunication fibres and it is able to withstand temperatures up to 80 °C. As the 20–100 °C range is expected for Cigéo application, it appears mandatory to consider alternative coating materials able to resist to higher temperatures. For such conditions, High-Temperature (HT) acrylate, polyimide coatings and metallic ones (Al, Au, Cu) can be used. Carbon layers deposited between the fibre glass and its coating also endure 100 °C and are incorporated to prevent the hydrogen diffusion from the surrounding environment into silica-based optical fibres [15]. As regards the various fibre prices, availability and optical performances, Andra selected HT-acrylate, polyimide and carbon-coated fibres. Au coating has been tested in France by EDF for Raman sensing in the 300–350 °C temperature range [16].

Samples were either purchased from the suppliers of Draka (Douvrin, France) and Fibercore (Southampton, UK) commercial off the shelf samples (COTS in the following), or ad hoc samples manufactured by the French iXBlue Photonics company (Lannion, France), allowing us to have a better control on the preform composition and its drawing conditions.

### 3.4. Optical Fibre Sensing Cables

On top of measuring principle and optical fibre type, selection of the sensing cable is of crucial importance. The external sheath ensures protection of the optical fibre (mechanical, chemical, hydraulic) and drives the strain transfer from the structure to sense/supervise to the optical fibre where sensing is performed. These two requirements are somehow opposite.

**Figure 4:**
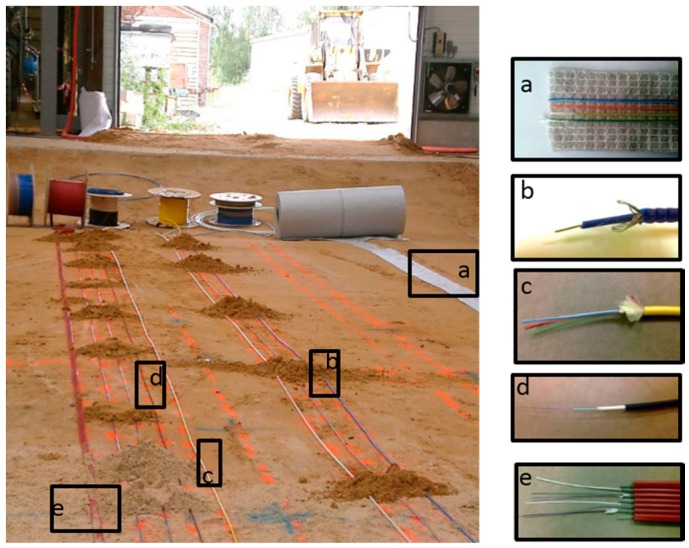
(Left) Picture of a one-to-one scale mock-up of dyke where several strain sensing cables (whose zooms are provided on the right) were tested.

Electricité de France (EDF, the French Electricity Company) and Andra have selected and tested several sensing cables, for soil monitoring, as illustrated in Figure 4 [17]. Similar comparative tests can be also performed for concrete and metallic structure monitoring [18,19]. The selected cables present advantages and drawbacks regarding handling (flexible and easy to install) and durability (metallic reinforced cable).

### 3.5. Strain and Temperature Metrological Benches

Several metrological benches were developed in order to impose controlled solicitation on optical fibre samples with several meter lengths (longer than the 1 m spatial resolution of most optoelectronic devices).

#### 3.5.1. Temperature Metrology

**Figure 5:**
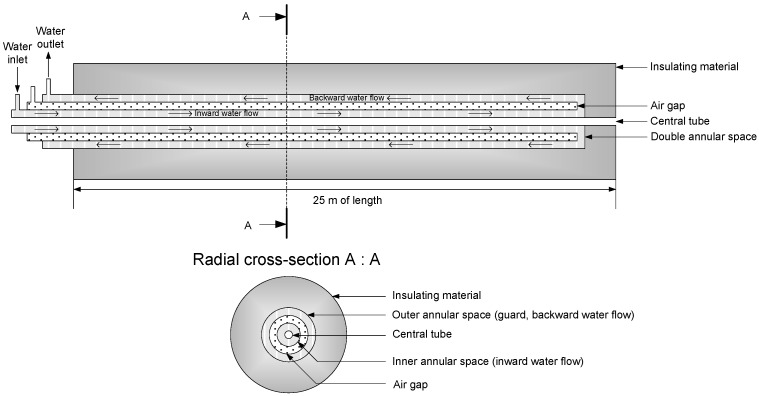
Schematic view of the 25 m horizontal furnace. Adapted with permission from [20]. Copyright 2016 IOP Publishing.

In collaboration with Andra and EDF, the Laboratoire National d’Essai (LNE, in Trappes near Paris, France) has built a dedicated facility in order to evaluate and to qualify distributed temperature sensing chains. Spatial resolution of the instrument is typically 1 m; the furnace had thus to be longer than 10 m for ensuring a spatially resolved measurement. As described in extended details in [20] and sketched in Figure 5, the horizontal furnace has a tubular configuration arranged as an assembly of five concentric stainless steel tubes. The central tube (internal diameter of 18 mm) can host 25 m of unrolled optical fibre. This furnace enables to perform temperature measurements with optoelectronic devices, by controlling the stability and homogeneity of the temperature around the fibre and by avoiding any mechanical stress. The fibre is fully free to move inside the tube, and free from any mechanical constraints, besides its own weight. The furnace temperature is controlled and stabilized by a water jacket. Enclosure is insulated to ensure a homogenous temperature field.

The 25 m long bench enables one to evaluate temperature sensing chain performances with temperature homogeneity and temperature stability over a duration of 20 h better than 0.1 °C and 0.05 °C, respectively [20].

#### 3.5.2. Strain Metrology

**Figure 6:**
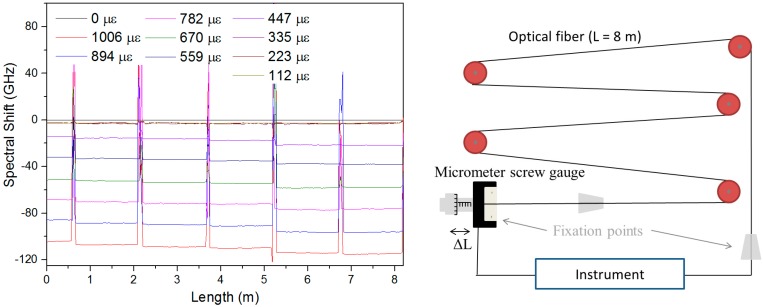
(Left) Example of distributed strain measurement obtained with TW-OTDR while a fibre is pulled at 10 different stress levels on the strain sensing bench (sketched on the right).

Andra and LabHC use two different techniques to evaluate strain sensitivities of optical fibres. The first design is based on pulleys [21]. Several meter-long fibre is supported by several pulleys, fixed at the two extremities; and strained over a length section L by applying a stepwise displacement ΔL using a micrometer screw gauge (Figure 6, right). The strain applied along the fibre is given by the relation: ε = ΔL/L, where L and ΔL are the length of stretched fibre and the displacement respectively. The total sample length was 8 m while the strain step was 100 µε in our case. As illustrated in Figure 6 left, the bench enables to create several controlled and homogeneous strain levels over more than one meter of fibre, between the fixation points.

**Figure 7:**
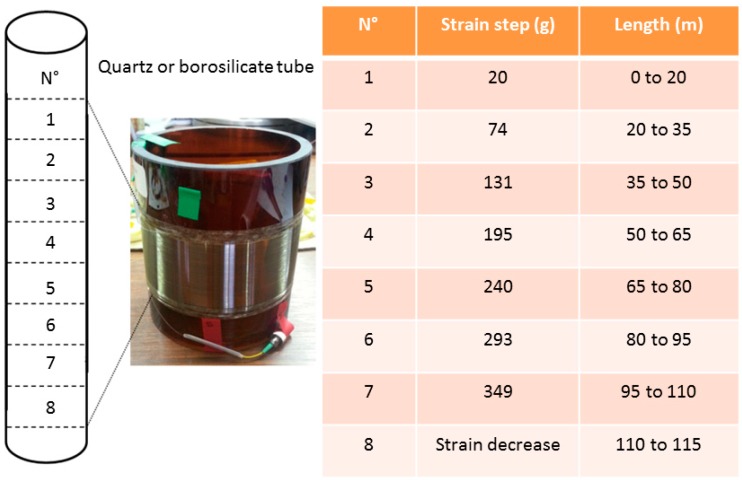
Schematic representation of the fibre coiling and picture of fibre sample strain steps. Borosilicate holder darkened under irradiation.

Another solution is to coil the Fibre Under Test (FUT) around a borosilicate [21] or a quartz [22] tube, by applying different controlled strains along the sample length. An illustration is reported in Figure 7 with a step strain every 15 m. Borosilica has a thermal expansion coefficient (TEC) identical to that of silica. As a consequence, such a setup permits one to verify in situ the impact of radiation on the fibre strain sensitivity coefficient. However, to transform weight into strain, the optical fibre Young modulus is required. It is proved to change with the optical fibre type and its influence is not negligible. This is why both strain calibration methods are useful.

**Figure 8:**
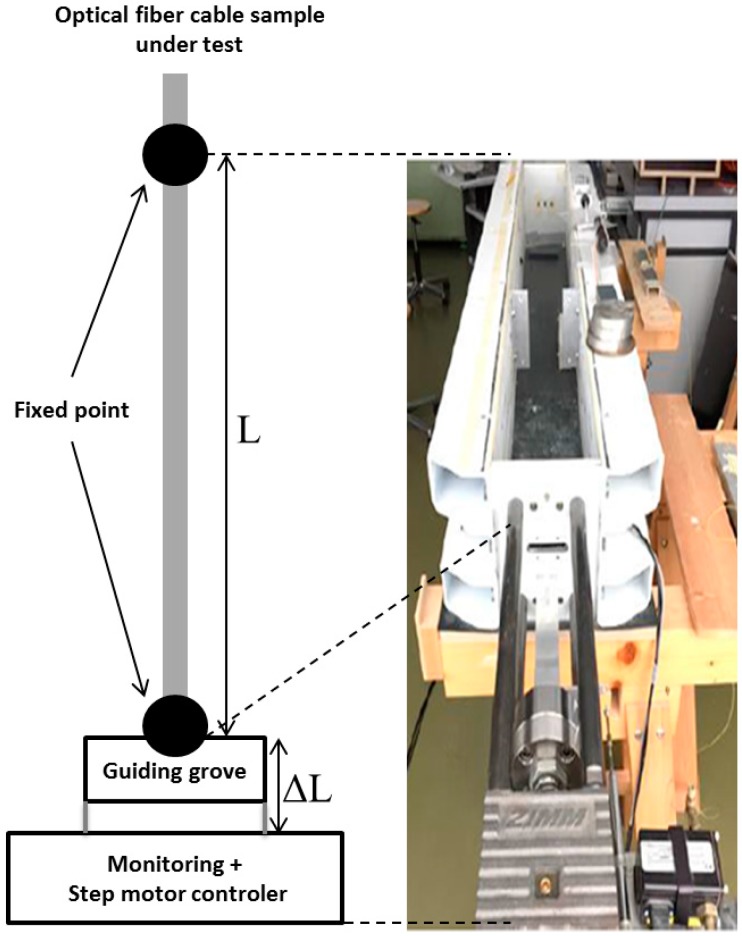
Schematic representation (**left**) of the bench used to determine fibre optic cables’ sensitivities to strain and pictures (**right**) from Marmota Company. Adapted with permission from [23]. Copyright 2016 Optical Society of America Publishing.

Finally, more robust bench designs must be used when sensing cables (opposite to optical fibres in primary coating), have to be characterized. Previous studies [23] demonstrated that the fibre strain coefficients of the Rayleigh and Brillouin responses are affected by the fibre packaging. Andra has used the testing bench of the Marmota company (Zurich, Switzerland), illustrated in Figure 8 to characterize the coefficients of the fibre cable. The fixation is designed in such way that it fixes the cable properly with a specific attention of no slippage effect between the cable and the clamp. The total length of the sample is about 5 m; the applied strain step can be controlled down to 100 µɛ while the room temperature is regulated.

### 3.6. Outdoor Tests

**Figure 9:**
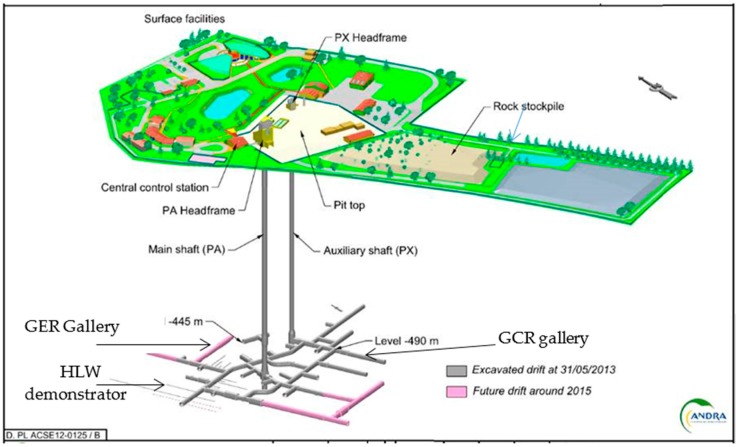
Schematic representation of the Andra’s URL and instrumented galleries (GER and GCR).

Andra has created an Underground Research Laboratory (URL, see Figure 9) at the beginning of 2000 in the town of Bure in Meuse district, at −490 m under the surface inside the Callovo-Oxfordian clay rock targeted for Cigéo project. Several one-to-one scale demonstrators of repository cells have been realized. Civil engineering designs are similar to those of the target application, but there is no radiation. 

**Figure 10:**
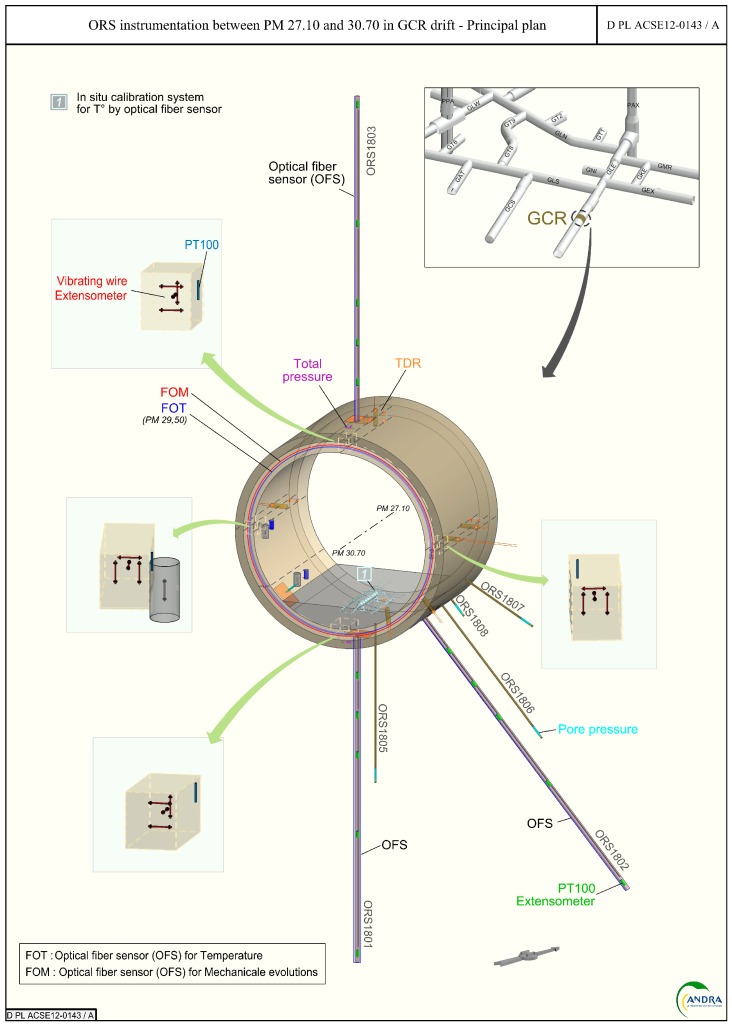
Schematic view of the monitoring section in the GCR gallery and pictures of implemented sensors, including optical fibre sensors.

Two ILW-LL cell monitoring demonstrators were realized [24,25]. They are tunnels of 5 m external diameter, with a shotcrete layer covering the host rock, a layer of poured concrete covering the shotcrete, basement construction, then slab and finally vault. In 2011 (respectively 2015), the “GCR” (resp. “GER”) gallery was dug according to the major (resp. minor) horizontal stress field. In these two galleries, several instrumented sections were implemented (see Figure 10), equipped with both classical and innovative sensor technologies (i) Fibre Optic Sensors (FOS), Vibrating Wire Extensometers (VWE), total pressure cells in the shotcrete; (ii) FOS, displacement sensors and pore water pressure in boreholes; (iii) FOS, VWE, Time Domain Reflectometry sensors (TDR) for water content measurement, permeability pulse sensor, total pressure cell. Such structures allow to test temperature and strain measurement solutions in real operating conditions. An instrumented HLW-LL demonstrator had also been realized and is depicted in details in [26].

### 3.7. Aging Tests

#### 3.7.1. Methodology for Aging Tests under Radiation

**Figure 11:**
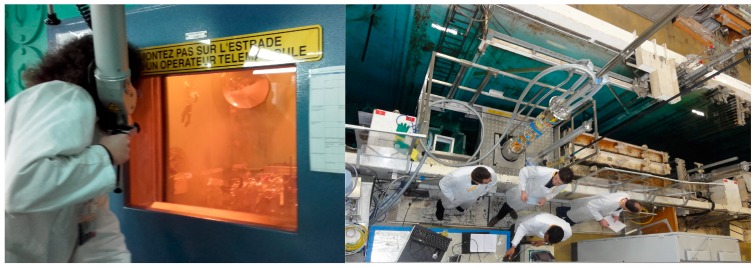
Picture of IRMA (**left**) and BRIGITTE (**right**) facilities used for irradiation of optical fibre sensors.

As it is usually the case for radiation environments, it is not possible to qualify the radiation response of the developed technologies in conditions representative of the application. At the external surface of the metallic liner of HLW repository cell, the total ionizing dose of 10 MGy will be deposited on the sensor in one century. As a consequence, it is mandatory to perform accelerated tests on the sensors and to extrapolate the expected radiation response of the system in the targeted environment. The strategy selected by Andra and LabHC consists in a three stage qualification. First the optical fibres have been irradiated passively (without monitoring) at the maximal dose expected for Cigéo: 10 MGy and the irradiated samples have been characterized post irradiation to evaluate the levels of permanent damages after such a huge dose. In a second step, the radiation response of the OFS are characterized online at a dose rate representative of the application but for a limited period (typically one or two weeks), providing information on the impact of such dose rate on their performances. Finally, a last campaign is achieved where the OFS performances are measured in situ at higher dose rate than the application and up to its maximal dose (here 10 MGy). This last campaign is considered as a worst case scenario. Indeed, it is well known that the degradation of the fibre in terms of RIA depends on both dose and dose rate and that for passive optical fibres, the degradation levels increase at higher dose rate and at fixed doses [27]. To perform this vulnerability study, Andra used the IRMA ^60^Co. source of the Nuclear Safety and Radiation Protection Institute (IRSN, Fontenay-aux-Roses, France) as this irradiation facility offers dose rates representative of the ones expected for the Cigéo project. The second used facility is the Big Radius Installation under Gamma Irradiation for Tailoring and Testing Experiments (BRIGITTE) ^60^Co facility of SCK-CEN (MOL, Belgium) that allows to obtain MGy doses representative of the total dose after one century of monitoring in Cigéo. These two irradiators are illustrated in Figure 11.

#### 3.7.2. Methodology for Aging under Hydrogen

The impact of the presence of hydrogen into optical fibres has been widely studied in the past, as loading germanosilicate fibres with this gas allows to increase its photosensitivity to ultraviolet laser light, opening the way to the photo-inscription of Fibre Bragg Grating [28]. As a consequence, tools exist allowing to saturate the fibres with hydrogen (or sometimes deuterium) gas in a few days, the kinetics of loading being adjusted by the pressure and temperature of the gas tank used for the treatment.

**Figure 12:**
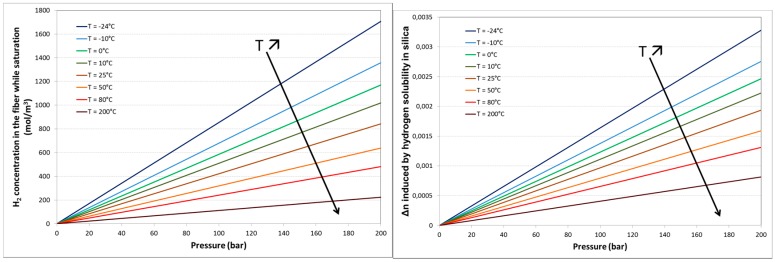
Theoretical values of the pressure and temperature dependence of the H_2_ saturation concentration in the fibre core center (**left**) and the related refractive index variation (**right**).

To investigate the effect of the presence of hydrogen molecules in the fibre core and cladding on the various technologies of OFS, the following procedure has been used. First, the various types of optical fibres (single-mode, multi-mode, different coatings…) have been treated under several conditions of temperature and pressure to reach different hydrogen saturation states. As soon as the fibre is removed from the gas tank, desorption process starts and the concentration of gas in the fibre core decreases with time. For a given fibre structure, known temperature and pressure conditions, the kinetics of gas absorption, the saturation level and the gas desorption kinetics can be calculated ([29] Figure 12). 

The presence of H_2_ molecules can be monitored (as well as an estimation of its concentration) through the well-known absorption bands of the hydrogen in the IR part of the spectrum [30].Then the impact of the hydrogen (or other gas) on the performances of an OFS can be evaluated for the whole range of hydrogen concentration (from zero to maximal initial concentration) by continuously monitoring the OFS performance during the long desorption period (typically a few day or weeks). In the framework of Andra research, existing tools and facilities of LabHC in Saint-Etienne, and iXBlue in Lannion have been used to perform such hydrogen loading in various experimental conditions.

### 3.8. Methodology for Radiation Sensing

Most of the studies about radiation effects on optical fibres are done to improve their radiation hardness to ensure the systems exploiting these fibres will be able to survive to the harsh environment of interest. However those studies also permit to identify radiation-sensitive optical fibres and it has been shown that their radiation response can be diversely exploited to monitor either the TID (total ionizing dose) and/or the dose rate. Optical fibres can then act as dosimeters. For some applications, the thermoluminescence or optically-stimulated luminescence (OSL) properties of optical fibres have been shown to be very attractive for post-irradiation dose measurements of various irradiation types with performances exceeding those of COTS Thermo-Luminescent Dosimeters (TLDs) [31]. Even more attractive, some fibres emit light (radioluminescence, RIL) when exposed to radiation, the RIL intensity being for adequate composition related to the dose rate or particle flux [32,33]. Most of the COTS scintillating fibres are based on polymer matrix [34], able to resist to low TID but recent progresses were done to achieve highly sensitive fibres in highly radiation resistant silica-based matrixes. However, these dosimeters exploiting the radiation induced emission of light are today mainly punctual sensors and more work is needed to design distributed measurements based on this RIE phenomenon in an unique fibre. Another class of fibre-based dosimeters exploit the dose dependence of the radiation-induced attenuation (RIA) in sensitive fibres such as those containing phosphorus, aluminium… Using reflectometry technique (OTDR, OFDR), the spatial distribution of the RIA can be monitored along a fibre exposed to irradiation and for each portion of the fibre, the deposited TID can be recovered from the RIA value (supposing the RIA vs dose dependence is known). Among the most promising fibres for such distributed dose measurements are the Phosphorus-doped ones as it was shown that the RIA levels at specific wavelength increase almost linearly with the dose and is quite insensitive to dose rate or temperature fluctuations [35].

### 3.9. Methodology for Hydrogen Sensing

Many types of H_2_ optical fibre sensors based on H_2_-sensitive materials such as WO_3_ [36], yttrium oxide (Y_2_O_3_) [37] or Pd compounds have been studied for several years. In contact with H_2_ gas, Pd forms an hydride PdHx (with x is a functions of the gas rate) leading to a variation of both the material refractive index [38] and lattice cell volume [38,39]. Efficient hydrogen sensing was achieved by using standard optical fibres (like G652 fibres used for telecommunications) with (i) Pd films deposited on the fibre cross-section [40] (ii) laid around the fibre [39,41]; (iii) around the core [42] or (iv) around tapered fibre [43]. However, these sensors are mainly dedicated to local gas detection, and they suffer from untimely deterioration in harsh environments and poor robustness [44]. They are mainly dedicated to local gas detection only.

Recently, Andra has demonstrated that H_2_ rich atmosphere leads to a very large shift of the backscattered Brillouin spectrum (around 21 MHz) in H_2_-loaded standard single-mode fibres [45]. Andra and Xlim intent to take advantage of this sensitivity for developing distributed hydrogen sensors. In this prospect, the Brillouin frequency shift induced by H_2_ diffusion in optical fibres need to be studied and finely quantified for dissociating H_2_ variation to others ones (temperature, pressure…). In contrast to most H_2_ sensing application that requires fast response time, slow variation of H_2_ concentration has to be monitored in Cigéo project which is compatible with the kinetic of H_2_ diffusion in silica fibre.

In this context, the methodology developed for sensing H_2_ is based on continuously recording simultaneous Brillouin and Rayleigh backscattering measurements during the H_2_ desorption process at ambient temperature and atmosphere. Following the methodology for hydrogen influence evaluation (cf. Section 3.7.2), optical fiber samples are H_2_ saturated in gas tank (at the iXBlue company in Lannion, France). Then, the fiber samples are sent to the Xlim Research Institute for desorption measurements.

Rayleigh and Brillouin backscattering measurements are realized simultaneously, on H_2_-loaded and pristine fibre samples with the instrument Neubrescope NBX-7020F (Neubrex Co. Ltd., Kobe, Japan) by tunable wavelength optical time domain reflectometry (resolution 1 GHz) and Brillouin optical time domain analysis (resolution 1 MHz) methods respectively [46]. Furthermore, a white light source and an optical spectra analyzer (ANDO AQ-6315A, Yokogawa, Tokyo, Japan) are used for measuring the attenuation of the peak at 1.245 μm induced by hydroxyl formation in the optical fibre [28]. This measure gives additional information on H_2_ concentration in the fibre during the desorption process. Contributions from acoustic velocity and refractive index variations induced H_2_ diffusion in the optical fibres are dissociated from Rayleigh and Brillouin backscattering measurements. To confirm the measurement of the refractive index variation, a fibre sample composed of a Bragg grating could be also inserted into the gas tank during H_2_ loading. An ASE light source and an optical spectra analyzer are then used for measuring the shift of the Bragg wavelength during H_2_ desorption, with a resolution of 50 pm. Experiments are realized with different H_2_ concentration at saturation that are obtained by H_2_ loading fibre samples with different temperature and pressure. All measurements in desorption are realized at room temperature (22 ± 1 °C), atmospheric pressure, and the fibre samples have been kept loose to minimize bias measurements.

Nevertheless, this methodology requires to send the H_2_ loaded fibre samples from the two French research partners, namely from Lannion to Limoges, which limits accurate measurements at the first desorption time, even if the desorption kinetic is strongly reduced by packing the fibre samples with ice blocks during the travel. In this context, the hydrogen setup developed by Mons University in Belgium (through MODERN2020 EU-project) that enables in situ measurements during H_2_ loading and desorption (with pressure and temperature continuously monitored) offers great prospects for pursuing our developments.

## 4. Results on Distributed Temperature Measurements

Andra plans to use Rayleigh and Brillouin scatterings for strain sensing after temperature characterization through Raman sensors. Based on its unique properties, Raman-based sensing is the Andra reference solution for temperature measurements.

### 4.1. Distributed Temperature Measurements Based on Raman Scattering

#### 4.1.1. Metrological Evaluation

**Figure 13:**
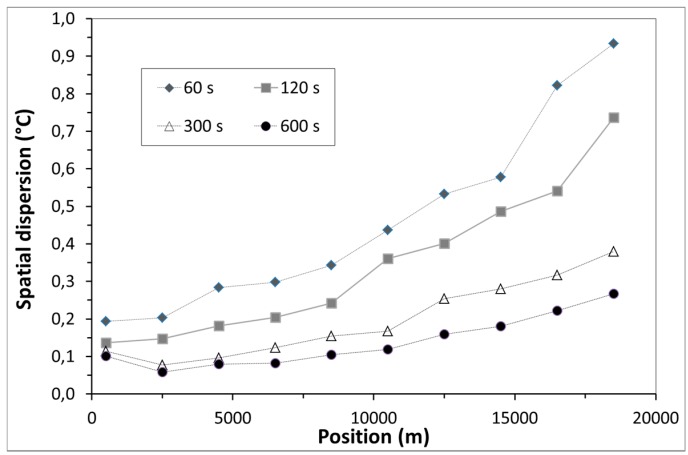
Measured temperature uncertainty (°C) as a function of the optical fibre length (m) at different averaging measurement times over at 23 °C and 1m spatial resolution.

The implemented methodology enabled to demonstrate the importance of wording, namely “spatial resolution” versus “sampling”. DTS response to a temperature variation step over one meter (spatial resolution typically claimed by the manufacturers) of sensing optical fibre corresponds to only 90% of the temperature step magnitude, whereas the full DTS response is obtained in fact for 10 m (the practical spatial resolution) of sensing optical fibre solicited by this temperature step variation [20]; depending on the instrument under test it can be 90% over 50 cm and 97% over 1 m [16]. Within METRODECOM project [47], Andra, EDF and LNE are performing a benchmark. Up to now, five commercially-available instruments were tested and compared and the results will soon be released [48]. Temperature uncertainty reaches 0.1 °C and degrades with distance along fibre, improves with measurement duration and spatial resolution (see Figure 13). Each instrument presents advantages and drawbacks to be confronted to the target application. For Cigéo monitoring, Andra presently prefers instrument specialized for short distance range, high spatial resolution and high Mean-Time Between Failure (MTBF).

#### 4.1.2. On-Site Evaluation

OFS were implemented in 2011 in the “GCR” gallery of the Andra URL. Fibre-optic Raman temperature monitoring systems were installed in three boreholes and in the concrete liner. The instrument has been working continuously for 5 years and must have been replaced in 2017. The second Raman device hold by Andra also failed after 5 years, despite exclusive use in laboratory conditions and several transportations. Similarly, Andra partners faced durability limitation: in the Mont Terri underground laboratory, Nagra had to improve the dust protection after failure of the Raman device during tunnel construction [49]. This is why MTBF is now an important criteria in device selection.

**Figure 14:**
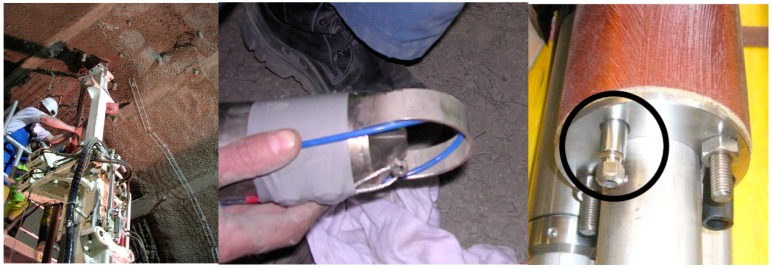
Pictures of distributed temperature system implementation inside vertical borehole (**left**). Example for a curvature guide (**middle**) and connector (**right**) to get the sensing cable through packers.

If the OFS cables are compressed or pinched, losses are created and measurement noise increases. What is more, as the bending losses are different at the Stokes and Anti-Stokes wavelengths, intensity ratio exploited to determine the temperature will be false (Equation (1)). Special tools were designed to limit the puncturing of the optical fibre, at both extremities of the borehole, that is to say the sealing and the far extremity (see Figure 14 [49]). 

To enable high-quality measurement provided by loop configurations (also required for radiation tolerance), the sensing fibre was installed with a U-shape. However, borehole diameter (8 cm and 13 cm) was smaller than the minimum curvature radius of the optical fibre. We took the risk and developed a curvature guide to optimise the position of the fibre. Measurements errors were noted anyway, and could be totally compensated subtracting the first measurement taken after the installation. This is why we conclude that the installation was successfully accomplished and Raman measurements are efficient as a relative temperature measuring system.

**Figure 15:**
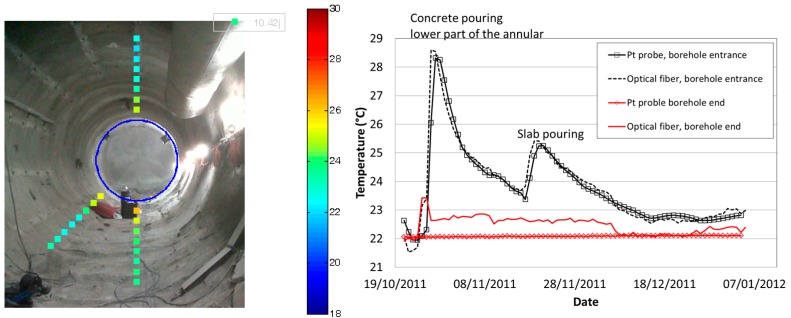
Distributed temperature measurement obtained with Raman OFS during concrete liner pouring (**left**) and comparison with collocated platinum probes as a function of time during concrete liner pouring (**right**).

Another difficulty that must be faced is how to extract the interesting measurements among the whole sensing line. From this point of view, distributed measurements are a powerful tool which might rapidly become time-consuming if not carefully configured. The device provides temperature as a function of a location in the optical fibre, which is never placed straight in the structure. To match Euclidien distance and curvilinear abscissa, it is of utmost important to realize a dynamic a map after installation, for instance, by heating or cooling remarkable locations. More industrial reference points can also be included in the sensing lines [50]. Under these precautions, as illustrated in Figure 15, distributed Raman systems produced good quality data over several hundred meters. This measurement was acquired during the concrete liner pouring and reveals heat propagation in the clay rock. Resolution and accuracy are satisfactory and in the same range as standard platinum probe temperature sensors (0.1 °C) placed nearby. The distributed temperature measurements along the fibre-optic cable reveal detailed insights into the spatio-temporal varying temperature field in the rock around the gallery. During the last four years, the most fragile parts proved to be the connectors on the multiplexor, which must be cleaned occasionally, unlike the sensing cables placed in the rock that resist very well [49].

#### 4.1.3. Accelerated Aging Tests: Hydrogen Influence

**Figure 16:**
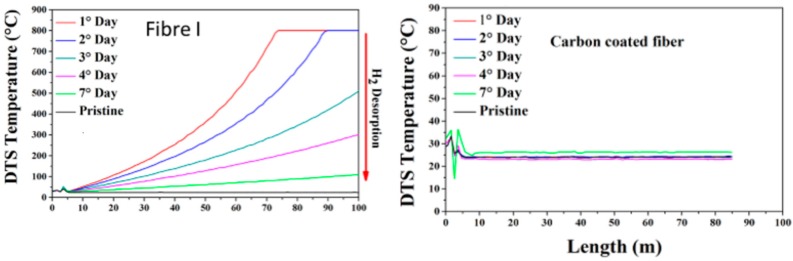
Influence of hydrogen exposure on temperature sensing based on Raman scattering for standard (**left**) and carbon (**right**) primary coating. Adapted with permission from [52]. Copyright IEEE 2015.

As detailed in [51,52], large temperature errors were observed along fibre samples exposed to hydrogen (80 °C with 202 bars of pressure for 62 h) then removed from the hydrogen tank, while the ratio between Stokes and Anti-Stokes Raman scattering (see Figure 16) was measured and interpreted in temperature following Equation (1). We also verify (Figure 16 right) that the presence of a carbon layer is efficient (and mandatory) to prevent hydrogen diffusion and degradation of the Raman distributed temperature sensors.

#### 4.1.4. Accelerated Aging Tests: Radiation Influence

**Figure 17:**
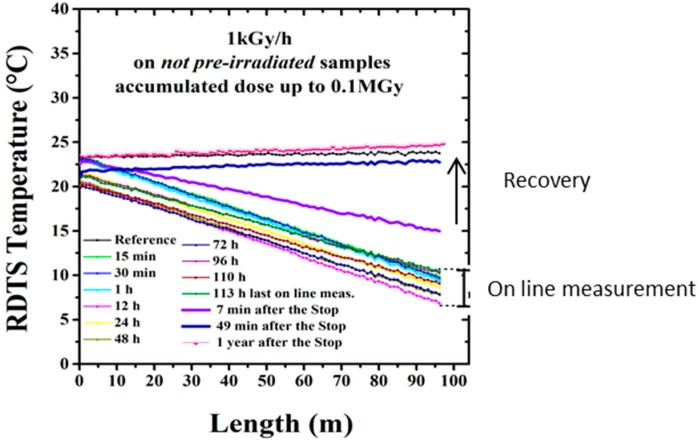
Distributed temperatures measurement performed, on Fiber I. Results obtained both during and after the γ-irradiation at 1 kGy(SiO_2_)/h dose rate. The red line indicates the first measurement from the start (our reference) and the dot line designates the room temperature (RT) at the beginning of the irradiation run. Copyright IEEE 2015.

Regarding radiation influence on temperature measurement based on Raman scattering, dramatic influence is observed (Figure 17): up to 50 °C error on a 100 m sample. Error increases with distance. Transient response significantly differs from post-mortem measurements, which enhances the importance of our methodology, with representative dose rate and cumulated dose, both post-mortem and on-line tests. Appropriate composition for the fibre (F-doped) allows reducing the amplitude of the temperature errors due to permanent effects of radiations but this is not sufficient to obtain acceptable resolution. The pre-treatments of the fiber, i.e., the ex situ pre-irradiation at 10 MGy(SiO_2_) reduces temperature measurement error (≤2 °C) [52].

Clear explanation of this phenomenon has been provided [51,52,53]. It is due to the differential attenuation at the Stokes and Anti-Stokes wavelengths created by RIA. α_S_ and α_AS_ differ rapidly under radiation, and the factor in Equation (1) will become unneglectable. To solve this wrong signal analysis, double-end measurements are promoted in literature [54]. New devices based on double-Raman probes are also to be considered [55]. Measuring RIA along the sensing device at S and AS wavelengths is another solution.

#### 4.1.5. Accelerated Aging Tests: Coupled Hydrogen and Radiation Influence

**Figure 18:**
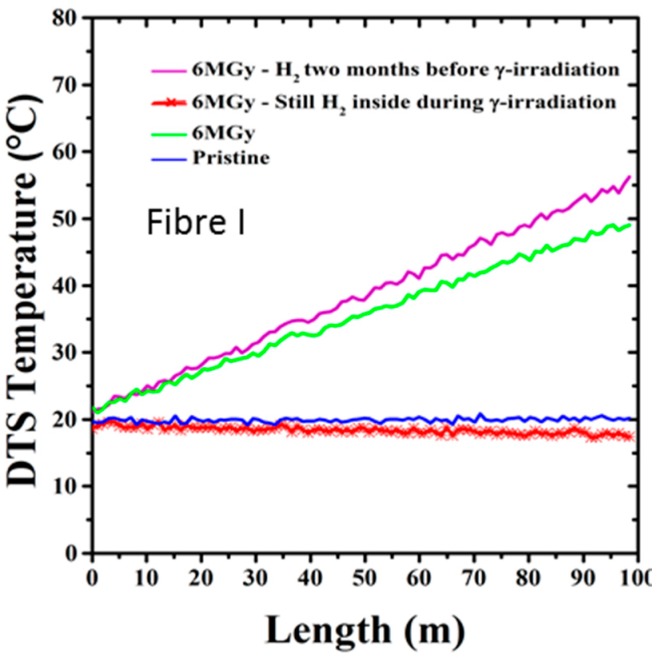
Distributed Temperature measurement performed on Fiber I, at room temperature. Results of pristine (light blue line), γ-irradiated at 6 MGy (red line), γ-irradiated at 6 MGy with H_2_ inside (green line) and γ-irradiated at 6 MGy two months after H_2_-loading (blue line) samples are shown.

Finally, it is worth notifying that hydrogen and radiation aging influence should not be considered independently. As illustrated in Figure 18 and detailed in [56], radiation impact is different whether the fibre has been previously exposed to hydrogen. We observe a meaningful negative effect of γ-rays on the sample H_2_-loaded two months before irradiation campaign and left to desorb naturally at ambient condition. Indeed, the response of the sensor is worse than without the H_2_ pre-treatment. We observe a temperature error exceeding the one of the 10 MGy irradiated sample (not reported in the figure). At the opposite, the sample irradiated in presence of H_2_ during the irradiation is only slightly affected by this combined treatment one month after the end of the irradiation.

#### 4.1.6. Conclusion for Temperature Measurements Based on Raman Scattering

Studies have shown that carbon coating is mandatory and fully satisfactory to perform temperature measurements in hydrogen rich atmosphere. Concerning radiation hardening, transient effects proved to be very important: post-mortem analysis cannot replace on-line testing. F-doped optical fibre (eventually pre-irradiated) is mandatory to limit RIA value and maximize the possible distance sensing range but is not efficient enough to reduce temperature measuring errors based on single-ended RDTS A solution is to implement other Raman scattering measuring configurations, such as double-end measurements (but sensing range is limited by a factor of 2) or to use innovative RDTS architectures.

We have shown that hydrogen and radiation effects are coupled. In Cigéo, there is also temperature to take into account. Andra has recently launched evaluation of coupled temperature influence (100 °C for Cigéo monitoring) on aging tests. Results will be released soon. Recent French study shows it should minimize radiation impact [16].

Regarding temperature measurements based on Raman scattering, TRL is presently estimated at 6 for Cigéo application: with all the listed precautions, OFS are ready to be implemented into the Pilot Phase.

### 4.2. Distributed Temperature Measurements Based on Loose Tube

Rayleigh or Brillouin scatterings are attractive for temperature sensing because both measurements can be performed in a single single-mode fibre. It fastens on-site implementation and thus reduces cost; a single device paired with a multiplexor could be used for both temperature and strain measurements, reducing costs.

Because of strain sensitivity, temperature measurement with these scatterings requires to isolate the sensing fibre from mechanical influence of the structure. This is called a loose optical fibre sensing cable. Andra has conducted several tests on “loose tubes” in the past, which revealed difficulties to isolate an optical fibre over very long distances [18]. Friction was unneglectable.

**Figure 19:**
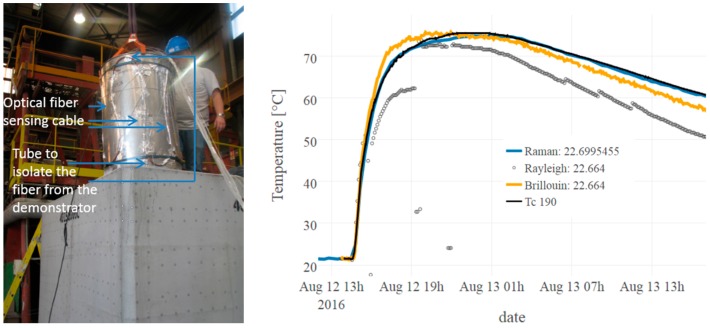
Loose tube implementation and test. (**left**) Picture of the emplacement of a demonstrator of primary waste package, instrumented with optical fibres (**right**) measured temperature on the waste demonstrator, obtained with Raman Rayleigh and Brillouin devices versus reference sensors.

Overlength must carefully be selected as a function of the application and the maximal temperature range. Implementation procedure must also be carefully adapted to ensure free dilatation. Meanwhile, the cable has to walk along and around the structure to provide a 3D characterization, but it is necessary to respect the radii of curvature to reduce friction. As detailed in [57], during a fire test performed at Efectis (Maizières-lès-Metz, France), to reduce the influence of differential thermal expansion, the cable was disconnected from the demonstrator. The selected process relied on curved copper tubes fixed to the structure. The sensing cables were threaded through these tubes. With such precautions, we managed to perform distributed temperature measurements with Brillouin and Rayleigh scattering acquired in a 50 m long loose tube. Brillouin and Rayleigh measurements were compared to Raman temperature measurements and to reference probes. Accordance was very good: Strain influence could not be distinguished. Slight discrepancies were observed on few locations (see for instance Figure 19). They attributed to the different acquiring spatial resolution (5 cm for Brillouin and Rayleigh versus 25 cm for Raman), while spatial gradients were important.

As a conclusion, distributed temperature based on Brillouin or Rayleigh scattering is now validated up to step 3 (over 4) of the qualification procedure for Cigéo monitoring. Compared to Raman scattering, implementation is more difficult, this is why it is not the reference solution. Aging tests will be presented in the following part, dedicated to strain sensing.

## 5. Results on Distributed Strain Measurements

### 5.1. Metrological Evaluation

From 2008 to 2012, Andra, EDF, Telecom-ParisTech and IFSTTAR have characterized many different single-mode optical fibres to evaluate their Brillouin scattering properties, both spectrum and: thermal and mechanical sensitivities. The goal was to evaluate whether the several Brillouin peaks could have significant sensitivities, in order to separate the two parameters to sense. Numerical modeling was also implemented; the influence of draw tension on the Brillouin peak position (up to −20 MHz/100 g) and its linewidth was quantified [58,59].

More recently, the strain sensitivity of the sensing cables were characterized. As detailed in [23], they may differ from the optical fibre’ sensitivity. For instance, following the methodology described in Section 3.2.5, Rayleigh strain coefficients of 0.79 µε^−1^ for the optical fibre versus 0.77 µε^−1^ for the fibre cable were obtained. For Brillouin scattering paired on two different strain sensing cable, we measured 0.0394 MHz µε^−1^ for the soft cable and 0.0465 MHz µε^−1^ for the reinforced cable [25].

These values were obtained on laboratory. Once embedded or attached to the structure to monitor, strain profiles measured in the optical fibre may differ from the actual strain in the structure, due to the shear transfer through the intermediate material layers between the optical fibre and the host material (i.e., in the protective coating of the sensing cable and in the adhesive).

The influence of the external sheath, both the geometry and the constitutive materials, has driven extensive evaluation in France these last years. In [60,61], EDF, in collaboration with Andra, and IFSTTAR have developed a methodology for the qualification of strain sensing cables in the host material. It relies on a numerical modelling of the cable, in which the mechanical parameters are calibrated from experiments. Once the transfer function is determined, measured strain into the optical fibre can be transformed into the strain in concrete. This work has been performed on a soft sensing cable, meant for embedment into concrete, illustrated in Figure 4. As detailed in [60], a four-point bending test was realized on a meter-long instrumented concrete beam. Crack openings were detected and quantified before it could be seen on external surface. It has also been shown the sensing cable could be attached on concrete rebars, there were no difference if placed between rebars. This eases and fastens real site implementation. Later, EDF developed an algorithm to automatically analyze crack evolution [62]. This process provided a precise map of cracks with the evolution of their amplitudes. Recent work focused on the transfer function of a sensing cable attached on the surface of structures [63]. It is of prior importance for old structure where sensors cannot be implemented inside materials anymore (which is not the case for Cigéo however Andra has application for the monitoring of short-lived waste repositories presently in exploitation).

### 5.2. On-Site Implementation

Truly distributed optical fibres strain sensing systems have been implemented in France in many civil engineering structures, including dykes [64], bridges, roads, tunnels and the nuclear power plant under construction, the EPR [65].

Andra and its industrial partners (IDIL Fibres optiques, EGIS, Cementys, Marmota, Solexperts and GTC) have implemented distributed strain sensing systems into concrete slab, during a building construction on surface [18], two concrete tunnels in the Andra URL (similar to a ILLL repository cell) and one horizontal tunnel with metallic liner (representing a HL cell demonstrator).

Obtained performances were reported in [19,24,25]. Specific one-site implementation difficulties were detailed in [49]. Similarly with temperature sensing, a key issue with OFS implementation was the pressure induced by clamps. Pinches generated high losses along the optical lines and poor signal to noise ratio. Unlike temperature sensing cables, where the fibre is protected from the host material, strain sensing optical lines suffered during the tunnel construction. The weakest part proved to be the interface between the sensing cables and communication cables. They could be repaired at the expense of total length change. Such slight changes (in the order of 20 cm of 500 m) in the total distance range and in the sensing area location proved to be very difficult to manage; On-line references are a promising solution [50].

**Figure 20:**
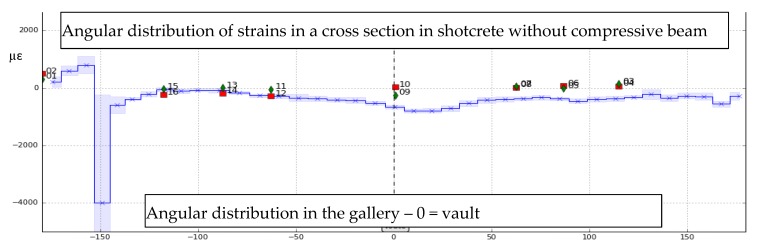
Strains measured by FOS (blue segments) and VWE (red squares for intrados location and green diamonds for extrados) around the cross section of the GER gallery (0° in vault and 180° in the bottom). FOS measured is the average stain over segments of 0.5 m. Blue line represents the moving window average of 12 h, with 95% confidence interval.

At this stage we showed that in the concrete liner, the tested strain optical fibre cables were sufficiently robust to tolerate construction conditions (with several dB losses in only few hundred meters). In GCR gallery, after 6 years more than 95% of the sensors still provide measurements, without any drift. What is more, strain measurements obtained in concrete liners with optical sensing cables are very in good accordance with collocated reference sensors, namely VWE [19,24]. In GER gallery, for FOS placed in the poured concrete layer, survival rate is 100%, after more than 1.5 year and good accordance is also obtained [25] (cf. Figure 20). Colocated optical lines were implemented, either spliced or equipped with connectors. Both survived to installation and provide accurate measurements. Since connected sensors allow faster operation than on-site splices, such configuration results much cheaper and will be promoted for future monitoring system implementation.

Feasibility of implementation in the shotcrete layer has been demonstrated in the GER gallery. FOS distributed strain measurements showed a good correlation with VWE’s [23]. However, as expected due to high strain in the shotcrete, the optical loop became too noisy after the first days, forbidding the use of stimulated Brillouin measurements. Strain measurements using Rayleigh instruments remained possible several weeks but also failed rapidly. New sensing cable that would tolerate larger strain and local pressure are under design in ITN-FINESSE project [66].

Finally, in Bure we also implemented strain optical fibre sensors in the HLW demonstrator [26]. An armored cable was placed at the interface between a metallic liner and the Callovo-Oxfordian clay. The cable endured very large stresses during liner pushing operations. However, the furthest extremity broke: at this location, the sensing cable was open inside a connection box, where the two optical fibres were spliced to provide a loop. It enhances the importance of the measuring device to be able to perform measurements on open loops and well as connected loops. Strain sensing cables implemented in the inner surface of the liner have been providing strain measurements for the last five years. Regarding durability of strain sensing devices [49], in Bure, a Brillouin device is running continuously since October 2011. We faced two returns to supplier over the three last years (one hard disk’s breakdown). The Rayleigh device test failed three times in Bure (contrary to several successful laboratory experiments and tests in HADES). We suspect the instrument to be totally incompatible with vibrations, created by permanent digging in several galleries of the Bure URL.

### 5.3. Aging Tests: Hydrogen Influence on Strain Measurements

Similarly with temperature sensing, single-mode fibres were hydrogenated. H_2_ rich atmosphere leads to large shifts of the backscattered Brillouin spectrum (around 21 MHz) in single-mode fibers loaded under 200 bars and 80 °C [45]. This corresponds to huge strain measuring errors, on the order of 400 µε. 

**Figure 21:**
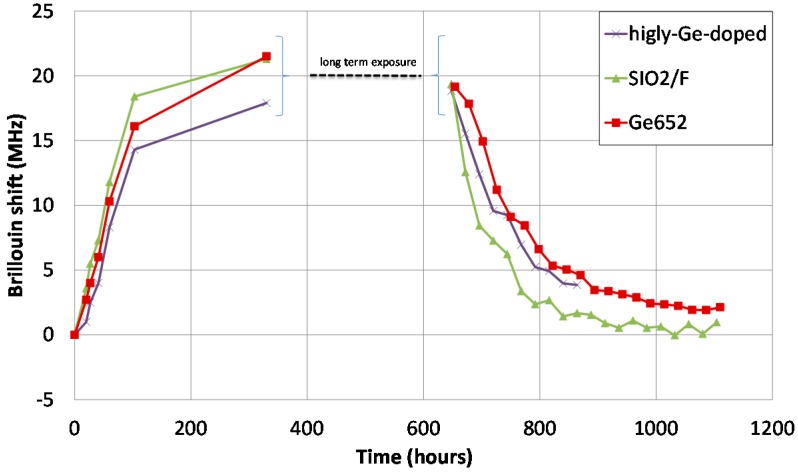
Brillouin frequency shift during the sorption and desorption period for CMS fiber (purple), Fluorine (green) and the most standard commercial G652 type fibre (red).

Optical fiber dopant type slightly changes the kinetics, not the Brillouin shift value (see Figure 21 and [67]). Carbon primary coating proved to be efficient to prevent hydrogen migration into silica [45]. This is why this primary coating is mandatory for strain sensing in hydrogen rich atmosphere, such as several repository cells of Cigéo (depending on radioactive waste type).

### 5.4. Aging Tests: Influence of Radiations

Radiations are known to influence the optical properties of silica-based optical fibres through ionizing or displacement damages. Depending on the irradiation conditions (nature of particles, dose, dose rate, temperature of irradiation), the observed changes in the optical and structural properties of the pure and doped amorphous silica of the fibre core can strongly differ. Regarding sensing applications, all OFS will be affected by the radiation-induced attenuation (RIA) that by decreasing the transparency of the fibre glass limits the sensing distance. Depending on the physical process used to functionalize the optical fibre as a sensor and of the sensor architecture, other phenomena can alter the OFS performance, such as radiation-induced refractive index change, glass compaction…

#### 5.4.1. On the Single-Mode Fibre Attenuation

**Figure 22:**
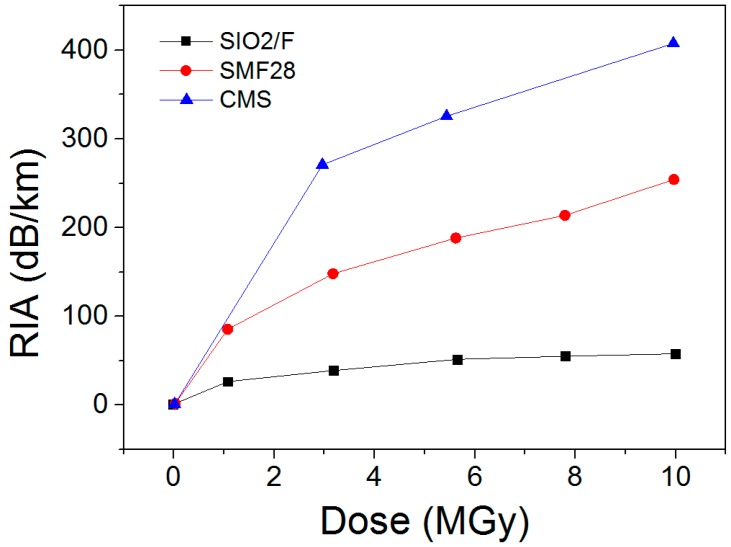
Measured RIA at 1.55 µm after different doses of irradiation from 1 to 10 MGy for three single-mode optical fibres: pure silica core fibre (SiO_2_/F), Telecom-grade germanosilicate (SMF28) and highly Ge-doped (CMS).

The amplitudes and kinetics of the RIA depend on many parameters that have been previously reviewed [68]. Among them, a crucial one is the composition of the fibre core and cladding that can totally change the RIA levels. Some compositions have to be avoided in radiation environments, such as the phosphorus doped or codoped optical fibres (except for dosimetry). Telecom-grade germanosilicate optical fibres present an intermediate radiation response that is acceptable for steady state kGy dose levels in the IR part of the spectrum but RIA is usually too high for applications at the MGy dose levels of CIGEO project, implying to select so-called radiation-hardened optical fibres that are usually designed either with fluorine or pure-silica core and fluorine-doped claddings [13]. Orders of magnitudes of the room temperature stable γ-ray RIA are provided in Figure 22 [69] at the wavelength of 1550 nm for a radiation hardened pure-silica core fibre and two germanosilicate fibres with moderate or high levels of Ge in their cores. If before irradiation, those fibres present attenuation below a few dB/km at this wavelength, the losses increase up to 400 dB/km after a 10 MGy dose. An important outcome of these studies is that at high radiation doses, the 1.3 µm RIA becomes below the one at 1.55 µm [69] but even by optimizing the fibre choice and operating wavelength, the OFS range will be reduced from several kilometres before irradiation to hundreds of meters after MGy irradiation.

#### 5.4.2. Radiation Impact on Brillouin Scattering

**Figure 23:**
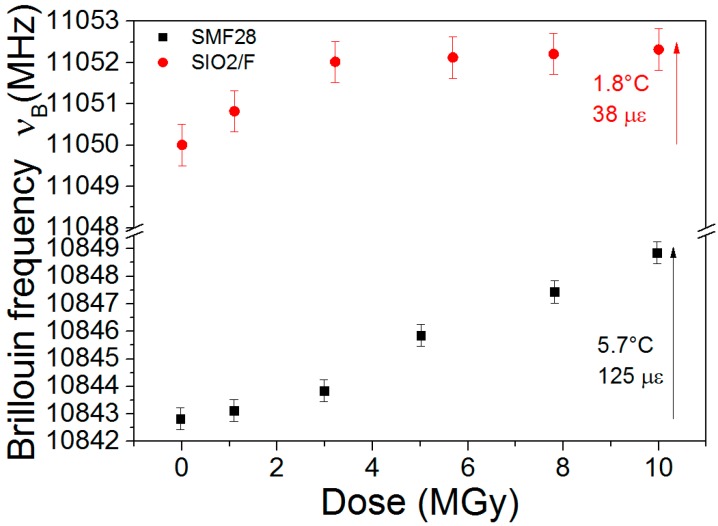
Measured Brillouin frequency shift for standard fibre with Ge dopants and F-doped fibre [69].

The radiation effects on Brillouin based sensors have been studied in the recent years in the frameworks of PhD thesis between Andra and LabHC. Radiations affect the performances of those sensors by two mechanisms. First, as for other technologies, the RIA limits the possible sensing range, reducing it to hundredths of meters at MGy doses for radiation-hardened optical fibres. Furthermore, radiations also induce a Brillouin frequency shift (RIBFS, see Figure 23) that causes a direct error on the measurement of the strain and or the temperature. Our studies shows that the amplitudes and dose dependences of this RIBFS depend on the fibre composition. Indeed, Figure 23 compares the RIBFS in a germanosilicate and a pure-silica core optical fibres. An outcome of our work is that selection of a pure or fluorine doped optical fibres appears the best choice to reduce simultaneously the RIA issues and the RIBFS. Doing this, Brillouin sensing remains possible for Cigéo application with limited expected errors on temperature (below 2 °C) and strain (below 40 µɛ) [70].

More recently, Andra and LabHC have also checked with on-line tests that transient effects were not important for Brillouin sensing, at least for moderate doses [21,51,71]. Based on promising results from [72], N-doped fibre has also been tested but it revealed less adapted to Cigéo than the qualified F-doped fiber [21].

#### 5.4.3. Radiation Impact on Rayleigh Scattering

The radiation effects on Rayleigh-based sensors have been studied in France in the recent years in the frameworks of PhD theses between Andra, LabHC and AREVA [21,73,74,75]. The same methodology as described in Section 3.7.1 was applied, either with OBR instrument [9] or TW-OTDR [10]. It was shown that the Rayleigh signature of various types of optical fibres (from radiation sensitive to radiation hardened) was not affected by irradiation up to 10 MGy, the temperature and strain coefficients being stable at 5%; this percentage can be improved by appropriate pre-treatment of the fibre coating (80 °C and/or pre-irradiation up to 3 MGy) to stabilize it for large irradiation tolerance. Furthermore, online testing up to 1 MGy dose and at various temperatures (from −40 °C to 75 °C) demonstrates the feasibility of temperature monitoring in such harsh conditions.

In conclusion, Rayleigh scattering seems then poorly sensitive to gamma rays. This is promising for Cigéo application, as the main remaining issue will be the RIA and its induced limitation in terms of sensing range.

### 5.5. Conclusion on Strain Sensing

Laboratory and in-situ tests were successful. Based on the presented aging tests, we are confident that Brillouin scattering performed in a carbon-coated F-doped fibre should handle 100 years of use under both radiation and hydrogen exposure. This conclusion has been reached based on the hypothesis that: (i) measuring device turns from 1.55 µm to 1.3 µm; (ii) post-mortem measurements at higher doses are similar or a worst case compared with the on-line influence at lower dose rate (iii) carbon primary coating remains efficient after radiation exposure. In a near future, on-line measurements must be performed to check that these results remain valid in case of on-line irradiation (the case of Cigéo application). It will also be important is to evaluate possible coupled impacts of both temperature, radiation and hydrogen on Brillouin and Rayleigh scatterings, similarly with the phenomenon observed for Raman scattering. Tests are on-going and results will be published soon.

Strain sensing will also be possible with Rayleigh measurements; on-line irradiations at high doses are on-going to check that the transient effects remain low. With these two qualified measuring methods (Brillouin and Rayleigh scatterings), a perspective is to perform both temperature and strain measurements in a single sensing cable, linked with the structure.

For Cigéo, the main perspective is to insert the qualified optical fiber into the qualified strain sensing cable (see Section 5.2); this work is on-going within MODERN2020 project. TRL is presently estimated at 5 for distributed strain sensing in the Cigéo application.

## 6. Results on Distributed Hydrogen Measurements

The Xlim research institute (CNRS and Limoges University, France) in collaboration with Andra, exploits hydrogen diffusion into silica and induced effects (see Figure 21) to realize distributed hydrogen measurements. This work initiated in 2014 was in first focused in understanding the diffusion mechanism of hydrogen inside silica optical fibre and its dependency to conditions (both temperature and pressure of the tank), and the geometry of the optical fibre. As illustrated in Figure 12, H_2_ concentration (in the fibre core) at saturation increases with larger pressure and lower temperature. This theoretical study is currently experimentally tested. It is of prior importance because it links optical properties such as variation of the refractive index, acoustic velocity) with external hydrogen quantities, the real sensing parameter.

This theoretical work has allowed us to investigate the physical mechanisms that provokes Brillouin frequency shift when H_2_ diffuses into silica. We have experimentally demonstrated that the Brillouin frequency shift is not only due to refractive index change with H_2_ concentration, but also to a variation of the acoustic velocity that increases with H_2_ concentration [29]. For example, we have measured a Brillouin frequency shift of 21 MHz induced by 1.7 %mol of H_2_ in silica that was obtained by exposing, until saturation state, a sample of standard single mode fibre (G652-type) to pure H_2_ gas at 25 °C with a pressure of 150 bars [45]. By decreasing the concentration of H_2_ at saturation in the fibre, we have measured a smaller shift of the Brillouin frequency (19 MHz for 1.14 %mol of H_2_ in silica) confirming the influence of H_2_ concentration on Brillouin frequency and its interest for H_2_ sensing applications. This smaller concentration at saturation was mainly obtained by increasing the temperature of the gas chamber (from 25 °C to 80 °C) as theoretically predicted and illustrated in Figure 12. Furthermore, the association of Brillouin and Rayleigh backscattered measurements allowed us to measure, during H_2_ outgassing period (13 days), a variation of the fibre core refractive index of the fibre core of 12 × 10^−4^ leading to a variation of the acoustic velocity by +6 m/s at saturation state.

In addition to these studies, specialty optical fibres have been developed to enhance hydrogen sensitivity. Andra and Xlim proposed to introduce Pd particles into the silica cladding of optical fibres in order to protect the sensing metal from harsh environments and for enabling distributed sensing of H_2_ gas along long lengths. Embedding Pd into fibres might improve the sensitivity and the response time of the distributed fibre gas sensor, by exploiting the mechanical strain induced by the crystal lattice expansion of Pd particles in contact with H_2_ gas. The fabrication feasibility of this kind of optical fibre has been demonstrated by exploiting an original fabrication process (based on powder technology) developed at Xlim [76].

**Figure 24:**
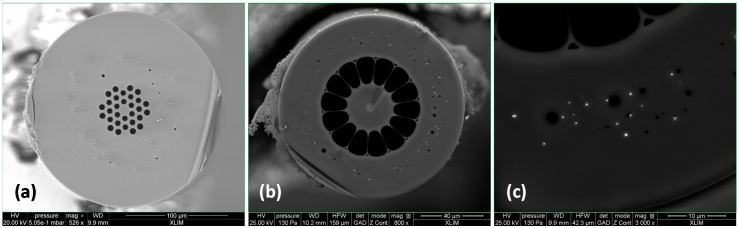
SEM (in the backscattered configuration) pictures of cross-sections of two optical fibres with Pd particles embedded into the silica cladding realized by the powder in tube process: (**a**) a pure silica core microstructured fibre; (**b**) a SiO_2_-GeO_2_ step index core with a microstructured cladding fibre; (**c**): zoom-in the cladding region of the fibre.

Different optical fibre topologies with Pd particles embedded into the silica cladding are presented in Figure 24. The fibres have been fabricated with lengths of several hundred meters and PdO concentration ranging from 0.01% to 5% mol (in addition to silica). Structural and microstructural characterizations of the preforms and fibres demonstrate that the fabrication process yields homogenous long lengths of fibres with metallic Pd particles randomly spread concentrically in the external cladding. However, the interest of Pd particules embedded into the silica cladding to enhance H_2_ sensitivity remains to be proved. Up to now, fibres were exposed to hydrogen. Then removed from exposure and measured when hydrogen was diffusing out of the fibre. It was difficult to start measurements at saturation, that is to say as soon as the fibres were removed from the hydrogen tank. The novel setup developed by Mons University will allow us to continuously measure Brillouin frequency shift during H_2_ loading and desorption. These on-line measurements will be performed in the coming months. Presently, we rate these developments at TRL 3 (laboratory stage only) level.

## 7. Results on Distributed Radiation Sensing

Several studies have been conducted to investigate the potential of OTDR or OFDR measurements of the radiation-induced attenuation (RIA) to monitor the distribution of TID along the optical fibre. Usually, those studies focused on the well-known radiation sensitive optical fibre such as those containing the Al or P dopants [14], but recently more radiation sensitive fibres (×10 times) have been reported in the framework of the DROID project from Universities of Perpignan and Nice [77]. A recent work was achieved by CERN on this topic combining commercial multimode P-doped fibres with COTS OTDR [78]. The authors demonstrate their ability to follow the dose distribution, with meter scale spatial resolutions and dose resolution down to 10–15 Gy, into the CERN High energy AcceleRator Mixed field facility, CHARM facility, that has an environment similar than the one of the Large Hadron Collider. One of the difficulties with such COTS solution is the trade-off between the mandatory high sensitivity to be able to detect the low dose RIA over typically one meter of the fibre and the range of measurements limited by the dynamics of interrogators. Preliminary study has been performed in Mons to investigate the potential of OFDR measurements of RIA offering higher spatial resolution (a few mm) than OTDR solution [14]. The authors demonstrate that such sensor can be used to monitor the dose increase up to 9 kGy with 15 cm resolution over about 1 m fiber length, the sensing length can be increased but only if lower dose levels are considered. In addition to these crucial issues, the integration of such solutions into facilities implies to calibrate the fibre RIA in function of not only the dose but also the temperature, the dose rate, the photobleaching effects that affect the RIA dose dependence [31]. Such a work is currently under progress in the framework of a PhD thesis between CERN and LabHC with the recent deployment of an OTDR-based dosimetry system exploiting the RIA at 1.55 µm in a P-doped multimode fiber at the Proton Synchroton Booster at CERN [79]. TRL is in the order of 3 (feasibility demonstrated in laboratory).

As a perspective, in 2017, the SURFIN project (a consortium between the Universities of Lille, Nice, Clermont Ferrand and Saint-Etienne (LabHC)) has been awarded for a grant by Andra within the framework of the French State “d'Investissements d’Avenir” program to investigate the feasibility of innovative dosimetry techniques to perform first punctual dose and dose rate measurements over a large range of doses, second to identify how the radioluminescence (RIL) or optically-stimulated luminescence can be exploited in a spatially-resolved dose and dose measurement and third how fiber materials can be adapted to discriminate between ionizing and non-ionizing radiations. Preliminary results have been obtained by University of Lille and LabHC with Cu and Ce-doped fiber materials, demonstrating their capability to monitor through RIL, X-ray [32] and proton [33] flux well lower than 1 Gy/s whereas being able to resist to more than 10 kGy accumulated doses.

## 8. Conclusions

We have presented the French state of the art distributed sensing systems, based on optical fibres, developed and qualified for the French Cigéo project, the underground repository for high level and intermediate level long-lived radioactive wastes. Four main parameters, namely strain, temperature, radiation and hydrogen concentration are currently investigated by optical fibre sensors, as well as the tolerances of selected technologies to the unique constraints of the Cigéo’s severe environment (temperature, hydrogen and radiation). A qualification method in four steps is being deployed: sensing chains are tested in laboratory, implemented into real structures, then evaluated in accelerated aging tests.

The performances of Raman, Brillouin and Rayleigh scattering have been studied for many optical fiber types, such as F-doped, Ge-doped, N-doped fibers and P-doped fibers, and several sensing cables. Succeful on-site implementation were reported; feedback enables to list several recommandations for tunnel monitoring with distributed OFS. The influences of both representative gamma radiation dose rates and total dose were estimated, as well as hydrogen influence. Cross-influences of hydrogen and radiations proved to be important. F-doped fiber (eventually pre-irradiated) and carbon primary-coatings are mandatory to limit RIA value and maximize the possible distance sensing range. The additional temperature influence on aging tests is still under evaluation, with the final objective to insert the selected optical fibre into the qualified strain sensing cable.

Strain and temperature distributed measurements based on Raman, Brillouin and Rayleigh scattering signatures now reach high TRL, suitable for implementation into the repository cells of radioactive wastes in the Cigéo Pilot Phase. Double-ended Raman-based sensing is the Andra reference solution for temperature measurements, with TRL6. Brillouin scattering proved to be appropriate for strain sensing (TRL5) but Rayleigh scattering is also studied as a back-up solution.

Future work will turn towards methodologies for fine measurement interpretations (from strain measurements to the real target parameters, such as tunnel convergence for instance). Metrology of optoelectronics is also driving much attention. Several optoelectronic devices endure failure or require maintenance after 5 years; methodology to characterize MTBF and interoperability of instruments are becoming a major issue.

Regarding distributed hydrogen and radiations sensing, developments presently remain at the laboratory stage. Promising solutions are proposed based on Radiation Induced Attenuation (RIA) responses of sensitive fibres such as the P-doped ones. While for hydrogen measurements, the potential of specialty optical fibres with Pd particles embedded in their silica matrix is currently studied for this gas monitoring through its impact on the fibre Brillouin signature evolution.

## Figures and Tables

**Table 1:** Main parameters to be monitored in the underground repository.

Parameters	Typical Range	Target Sensitivity	Spatial Homogeneity
Temperature	[20–90 °C]	±0.1 °C	20 cm
Displacement	+0.5 mm/m to 2.5 mm/m	1 µm/m	10 cm
Strain in concrete	10 μm/m	3 μm/m	10 cm
Concrete Crack	Threshold for openings: 200 μm		10 cm
Gap evolution inside the cell	10 mm (in 100 years)	0.5 mm	1 m
Hydrogen	[0–4%] sensitivity of 500 ppm [4–10%] sensitivity of 1%	100 ppm<1%	3 m (ILW-LL waste package size) ~1.5 m (HL waste package)
Gamma radiation	0.1–1 Gy/h Total cumulated dose 10 MGy (100 years)	50 mGy	~1.5 m (HL waste package)

**Table 2:** Typical performances of distributed temperature and sensing systems based on Rayleigh, Brillouin and Raman scatterings in optical fibres.

Scattering	Rayleigh		Brillouin		Raman
Process	Elastic		Inelastic		Inelastic
Optical fibre type	Single-mode		Single-mode		Mainly multi-mode
Measuring principle	OFDR	TW-COTDR	BOTDR	BOTDA	R-OTDR
Acces to fibre	Single end		Single end	Loop configuration	Single end
Maximal distance range	70 m	20 km	30 km		30 km
Best spatial resolution	10 mm	20 cm	1 m	10 cm	1 m
Temperature sensitivity	*C_T_*^R^ = −1.5 GHz/°C		1 MHz/°C		0.1 °C
Strain sensitivity	*C^R^*_ɛ_ = −0.15 GHz/µɛ		0.05 MHz/µɛ		Not sensitive
Measurement Uncertainty	0.1 °C	0.5 °C	5 °C	1 °C	0.01 °C
Measurement duration	10 s	10 min	10 min		1 min
Optical budget	70 dB	10 dB	10 dB		10 dB

**Table 3:** Optical fibers selected for the ANDRA qualification process.

Type	Core	Cladding	Coating	Names
SM-Ge	Ge-SiO_2_ (28 wt %)	Pure silica	Acrylate	CMS
SM-F	F-SiO_2_ (0.2 wt %)	F-SiO_2_ (1.8 wt %)	Acrylate	SIO2/F
MMF	Pure Silica Core	F-SiO_2_	Acrylate	Fibre I
MMF	P-SiO_2_	Doped-SiO_2_	Carbon	HDR MMF
